# Development of a Distributed Crack Sensor Using Coaxial Cable

**DOI:** 10.3390/s16081198

**Published:** 2016-07-29

**Authors:** Zhi Zhou, Tong Jiao, Peng Zhao, Jia Liu, Hai Xiao

**Affiliations:** 1 School of Civil Engineering, Dalian University of Technology, Dalian 116024, China; jiaotong@mail.dlut.edu.cn (T.J.); zhaopengdut@sohu.com (P.Z.); gabrielleliu@163.com (J.L.); 2 State Key Laboratory of Coastal and Offshore Engineering, Dalian University of Technology, Dalian 116024, China; 3 Electrical and Computer Engineering Department, Clemson University, Clemson, SC 29634, USA; haix@clemson.edu

**Keywords:** coaxial cable, crack, distributed sensor

## Abstract

Cracks, the important factor of structure failure, reflect structural damage directly. Thus, it is significant to realize distributed, real-time crack monitoring. To overcome the shortages of traditional crack detectors, such as the inconvenience of installation, vulnerability, and low measurement range, etc., an improved topology-based cable sensor with a shallow helical groove on the outside surface of a coaxial cable is proposed in this paper. The sensing mechanism, fabrication method, and performances are investigated both numerically and experimentally. Crack monitoring experiments of the reinforced beams are also presented in this paper, illustrating the utility of this sensor in practical applications. These studies show that the sensor can identify a minimum crack width of 0.02 mm and can measure multiple cracks with a spatial resolution of 3 mm. In addition, it is also proved that the sensor performs well to detect the initiation and development of cracks until structure failure.

## 1. Introduction

Cracking is a major sign of structural aging and damage, which shortens the integrity and service life of structures. While the width of cracks for most ordinary reinforced concrete (RC) structures in civil engineering are over 0.2–0.4 mm, the service life of the structure can be severely shortened due to the erosion of inner steel rebar under harsh environments. Moreover, structures may be damaged if the cracks reach 1–2 mm. Therefore, crack monitoring is essential to guarantee the safety of structures. Currently, many approaches have been developed for crack monitoring, such as ultrasonic methods [1], acoustic emission [2], infrared thermography [3], impact-echo [4], large area electronics [5], etc. All of the techniques mentioned above show good performance of crack detecting, however, they struggled to be applied in practical engineering due to the installation difficulties and vulnerability in the long-term harsh environment. Recently, the optical fiber sensing technology, such as the Bragg grating, the Michelson white light interferometer, and Brillouin scattering have been widely investigated for crack detecting [6,7,8]. However, all of the fibers in the above approaches are silica fibers which are brittle (their elongation rates are only about 1%) [9], which make it easy to break in real applications, and only small cracks can be monitored. Therefore, the brittleness of silica fibers limits the application of the approaches above.

To overcome this disadvantage, coaxial cable, which has a high elongation rate (more than 20%) and works similarly as an optical fiber since they share the same fundamental physics governed by the same electromagnetic (EM) theory, was attempted to monitor the cracks. Lin et al. [10,11] investigated the feasibility of using an embedded commercial coaxial electrical time domain reflectometry (EDTR) cable to detect crack damage in a reinforced concrete beam subjected to a three-point bending load. Although the ETDR signal response waveforms of the sensing cable were able to capture the location of the crack damage site and indicate the relative magnitude of the crack opening, the commercial ETDR sensing cable has low signal-to-noise ratio and low sensitivity. Afterward, a prototype cable was designed and fabricated by Lin et al. [12,13], using rubber as an insulator of the cable instead of polyethylene or Teflon materials employed in commercial cables. According to the calibration tests conducted by Lin et al. [12,13], the prototype cable is approximately ten times more sensitive than commercial cables. The commercial and the prototype ETDR sensing cable are all designed according to the geometric change of the cables, which is low in sensitivity due to the lower sensitivity of the cable impedance to the cross-sectional change. To improve the sensitivity, Chen et al. developed two designs of coaxial cable sensors based on the change in topology of the cable’s outer conductor under strain conditions. One was designed by wrapping a dielectric rubber tube spirally with copper tape, which is the outer conductor of the cable [14]. The other was reconstructed by using Teflon as the dielectric layer and a steel spiral as the outer conductor. The steel spiral can slide along the Teflon surface under strain conditions [15]. These sensors are claimed to be more sensitive than commercial products under applied loads. The uniformity of the sensors, which are mainly associated with their fabrication process, is significantly improved by the plasma-spray coating technique [16]. The topology-based cable sensors have shown many advantages in practical applications [17,18]. However, the spirally-wrapped outer conductor of the sensors can easily lead to loose contact and substantial signal attenuation, which make it easy to break during the installation process and cannot apply to long distance monitoring.

In this study, an improved topology-based cable sensor with a shallow helical groove on its external surface of the outer conductor is developed. The inter-relationship among various design parameters is explored through numerical simulations. The following calibration tests investigate the sensor performance for crack detecting. Finally, a feasibility study is also conducted to demonstrate the utility of the sensor in practical applications.

## 2. Sensing Mechanism of ETDR

Coaxial cable is a microwave transmission line. It consists of an inner and outer conductor sandwiched by a tubular insulating layer with a high dielectric constant. Based on the transmission line theory, the characteristic impedance of a cable is the ratio of the voltage to the current of the guided wave. It is related to the distributed parameters, including inductance, capacitance, resistance, and conductance of the cable. Using an equivalent circuit model, their quantitative description can be derived as Equation:

(1) $$Z_{0}=\frac{U_{+}\left(Z\right)}{I_{+}\left(Z\right)}=-\frac{U_{-}\left(Z\right)}{I_{-}\left(Z\right)}=\sqrt{\frac{R+j\omega L}{G+j\omega C}}$$

where $Z_{0}$ is the characteristic impedance, $U_{+}\left(Z\right)$ and $U_{-}\left(Z\right)$ are the incident and reflected voltage at point Z, respectively, $I_{+}\left(Z\right)$ and $I_{-}\left(Z\right)$ are the incident and reflected current at point Z, respectively, L, C, R, G represent the inductance, capacitance, resistance, and conductance, respectively, and ω is the angular frequency of EM wave.

In general, the propagating signal in the coaxial cable is high frequency EM waves (>100 kHz), so R<<ωL, G<<ωC and, thus, the characteristic impedance can be simplified as:

(2) $$Z_{0}=\sqrt{\frac{L}{C}}$$

**Figure 1:**
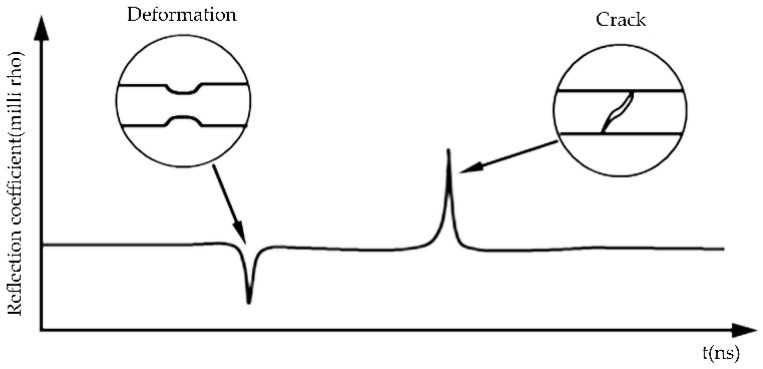
Schematic illustration of the waveform of a coaxial sensing cable under loading.

Equation indicates that the characteristic impedance of the sensing line has a distributed feature. For an ideal coaxial cable, the EM wave propagating along the sensing line will not be reflected since the characteristic impedance is constant and uniform. If there is a load-induced local deformation of the cable’s cross-section, or if a change in topology of the cable’s outer conductor happens, an impedance discontinuity will be present along the sensing cable, which, in turn, results in the signal reflection at the point of discontinuity. As shown in Figure 1, the reflected step signal carries the information about the location and severity of the discontinuity. The position of the discontinuity, D measured from the monitoring point can be determined by:

(3) $$D=\frac{1}{2}v\cdot t$$

where v is the propagation velocity of the signal along the sensing cable, and t is the transit time of the signal propagating forth-and-back from the monitoring point to the discontinuity point.

The severity of the discontinuity is related to the reflection coefficient, which is defined as the ratio between the reflected voltage to the incident voltage. The reflection coefficient, ρ, can be expressed as:

(4) $$\rho =\frac{U_{r}}{U_{i}}=\frac{Z^{\prime }-Z_{0}}{Z^{\prime }+Z_{0}}$$

where $U_{r}$ and $U_{i}$ are the reflected voltage and the incident voltage, respectively. $Z^{\prime }$ and $Z_{0}$ are the characteristic impedances at the discontinuity and along the uniform transmission line, respectively.

According to the principle mentioned above, coaxial cable can be developed to detect both the location and width of a crack at any point in a structure through the ETDR technique.

## 3. Design and Fabrication of the Prototype Sensor

**Figure 2:**
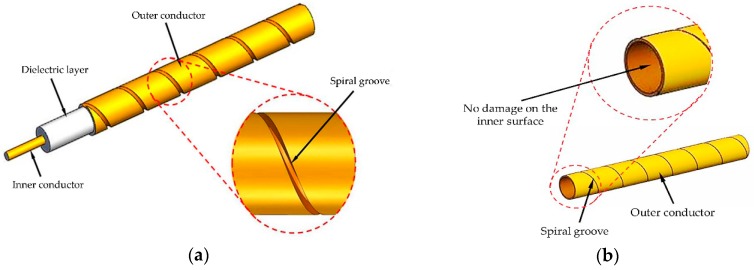
Sketch map: (**a**) Prototype crack sensor; (**b**) details of the inner surface of the sensor.

**Figure 3:**
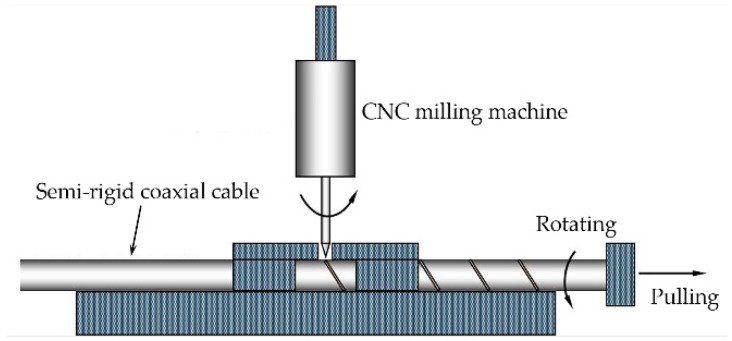
Prototype sensor fabrication process.

A new type of coaxial cable sensor for crack detecting is designed based on the ETDR sensing principle in this part. As shown in Figure 2a, the prototype sensor is a topology-based sensing cable with a shallow helical groove on the external surface of the outer conductor of a semi-rigid coaxial cable, whose outer conductor is a seamless copper tube. To precisely control the shape and depth of the helical grooves, a computer numerical controlled (CNC) milling machine capable of pulling, rotating, and notching grooves is used to fabricate the sensor. In the process of fabrication, a semi-rigid coaxial cable is mounted on the CNC milling machine with the milling depth set properly. Then the grooves are produced continuously on the outer conductor by a milling cut while the cable rotates and moves along its axis. The key factor in the fabrication is to ensure that the shallow helical grooves cannot cut through the outer conductor, but could be separated easily under loading (Figure 2b). Figure 3 shows the schematic of the sensor fabrication process.

**Figure 4:**
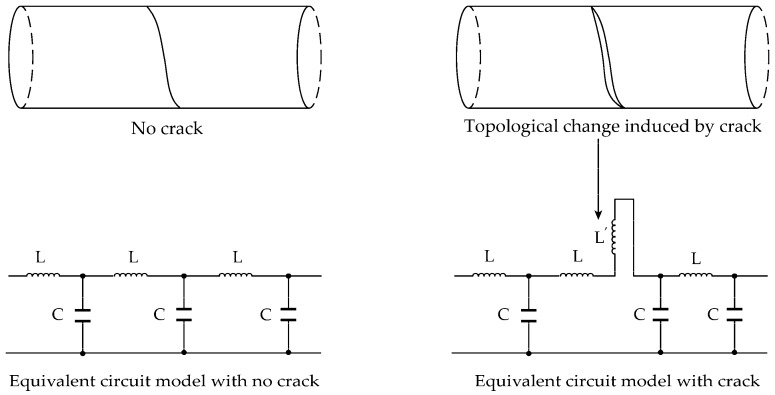
Equivalent circuit model of the prototype sensor.

For the prototype sensor applied to crack monitoring, the outer conductor is closely connected with its inner surface before crack generation. When a crack on the structure forms and grows, the shallow spiral grooves will easily separate under mechanical loading, resulting in the change of the current flow path along the outer conductor. Based on the equivalent circuit model, the distributed inductance is related with the voltage and current, in accordance with Equation. Thus, the change in current flow path will introduce a lumped inductor (Figure 4). According to Equation, an “impedance discontinuity” will be formed because the impedance at the location of the crack changes from $\sqrt{L/C}$ to $\sqrt{(L+L^{\prime })/C}$. Thus, a portion of the incident wave will be reflected when it encounters this discontinuity. Following the ETDR sensing principle, the locations of cracks are determined by the arrival time of the reflected signal while its amplitude represents the degree of the cracks. Thus, the proposed sensor embedded in the structure can detect both location and width of the crack. Equation indicates that the spatial resolution for crack detecting is determined by the rise time of the incident impulse signal and the bandwidth of the sensor. For a certain rise time of the incident impulse signal, the larger the transmission bandwidth of the sensor corresponds to a higher spatial resolution.

(5) $$L=\frac{\int U(t)dt}{I(t)}$$

(6) $$\Delta L_{\min}=\frac{1}{2}v\sqrt{{T_{step}}^{2}+{\left(0.35/BW\right)}^{2}}$$

## 4. Numerical Simulations

To fully understand the correlation between the sensor performances and their major design parameters including crack width, spiral grooves interval and rise time of incident impulse signal, the proposed sensors were numerically simulated using a full-wave numerical solver (Ansoft HFSS). In the simulation, several 100 mm-long sensors were modeled with an outer conductor diameter of 0.5 mm and the dielectric layer diameter of 1.7 mm. The relative permittivity of the dielectric material was set to be 2.1, and the characteristic impedance was 50 Ω. When removing a portion of several helix wires, an air gap can be created to represent a partial separation of spirals caused by the cracks applied on a sensor. The ETDR response waveforms under the excitation of a Gaussian pulse were obtained by integrating the voltage waveform over time. Herein, crack width is characterized by the separation width of the spiral grooves.

**Figure 5:**
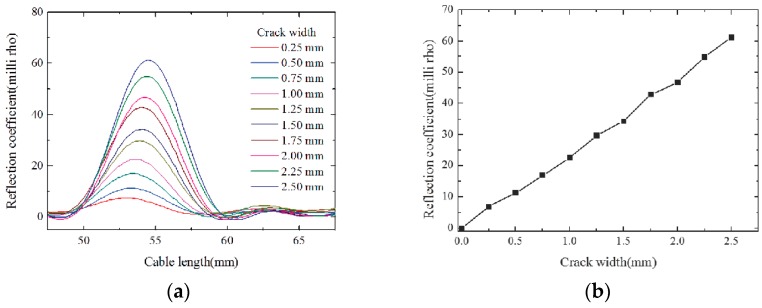
Results of simulations: (**a**) reflection waveform; and (**b**) the correlation between reflection coefficient and crack width.

**Figure 6:**
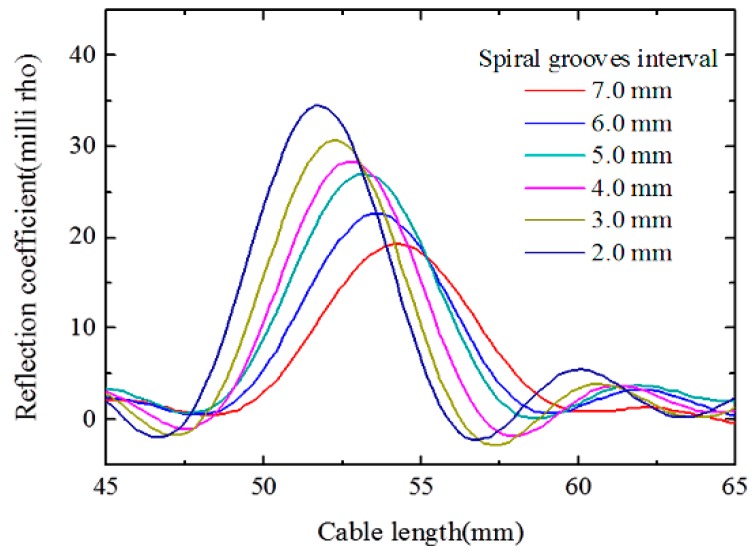
Responses of the sensor under various intervals of spiral grooves.

**Figure 7:**
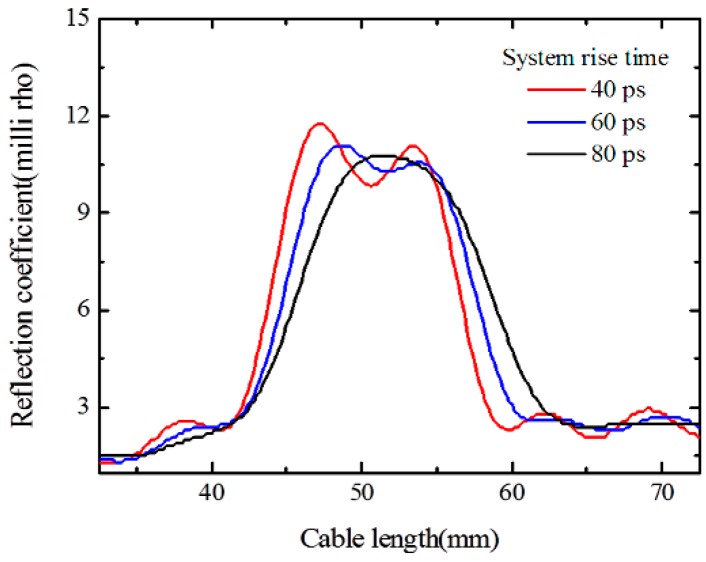
Correlation between the spatial resolution and the system rise time.

Figure 5a shows the simulated ETDR waveforms of the sensor with a fixed spiral separation length of one turn and a spiral groove interval of 6 mm, and a different crack width from 0.25 mm to 2.5 mm with an increment of 0.25 mm. Figure 5b illustrates the relationship between the peak values of the reflection coefficient and the crack width. Simulation results demonstrate that the peak values of the reflection coefficient increase almost linearly within the crack width. For the simulated sensors with a fixed spiral separation length of one turn and a crack width of 1.0 mm, when the spiral groove interval changes from 2.0 mm to 7.0 mm with an increment of 1.0 mm, the peak values of the reflection coefficient gradually decrease as shown in Figure 6. It indicates that the sensitivity of the proposed sensor is related to the spiral groove interval, and the higher peak value of the reflection coefficient corresponds to a smaller interval of the spiral groove. Therefore, the proposed sensor with a smaller interval of spiral grooves is expected to perform with better sensitivity. To investigate the effect of the rise time of the incident impulse signal on the spatial resolution, a sensor with a fixed spiral separation length of two turns and a spiral groove interval of 6 mm, as well as a crack width of 0.5 mm was simulated. According to Figure 7, there are two clear peaks in the response waveforms when the rise time is 40 ps, however, the two response peaks become gradually indistinct with the increase of the rise time. It means that the spatial resolution is related to the rise time of incident impulse signal, and a smaller rise time can lead to a higher spatial resolution.

## 5. Sensor Calibration

**Figure 8:**
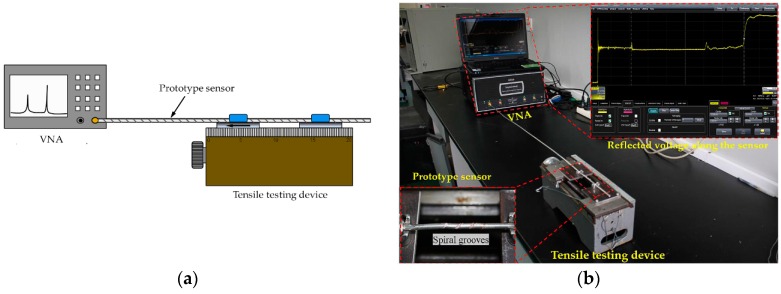
Setup of sensor test system: (**a**) schematic representation; and (**b**) overview.

In this part, the crack detecting capability of the prototype sensor was investigated using a test system, which consists of a vector network analyzer (VNA), a tensile testing device and a prototype sensor. The overall setup of the experiment and their schematic representations are shown in Figure 8a,b, respectively. The VNA used in the system has a 40 GHz measurement bandwidth and launches the Gaussian pulse with a rise time of 6 ps. The tensile testing device, capable of measuring micro-displacements, can elongate the cable with the minimum stepping movement of 0.01 mm. The commercial semi-rigid SFT-50-3 coaxial cable was selected to fabricate the sensor following the method of the above third part. Table 1 lists the geometric configuration and electrical properties relevant to the sensing characteristics of the sensor.

### 5.1. Sensitivity Test

In theory, the proposed sensor has the distributed detecting ability of cracks because any separations along the spiral groove resulting from cracks will introduce the reflected signal on the EDTR waveform. Since the objective of this test is to investigate the influences of crack width and the spiral groove interval to the reflected coefficient, the sensors tested were made of only one turn of the spiral separation. Thus, two types of prototype sensors were considered in the sensitivity test. The main difference between the two sensors is their spiral groove interval as presented in Table 2.

**Figure 9:**
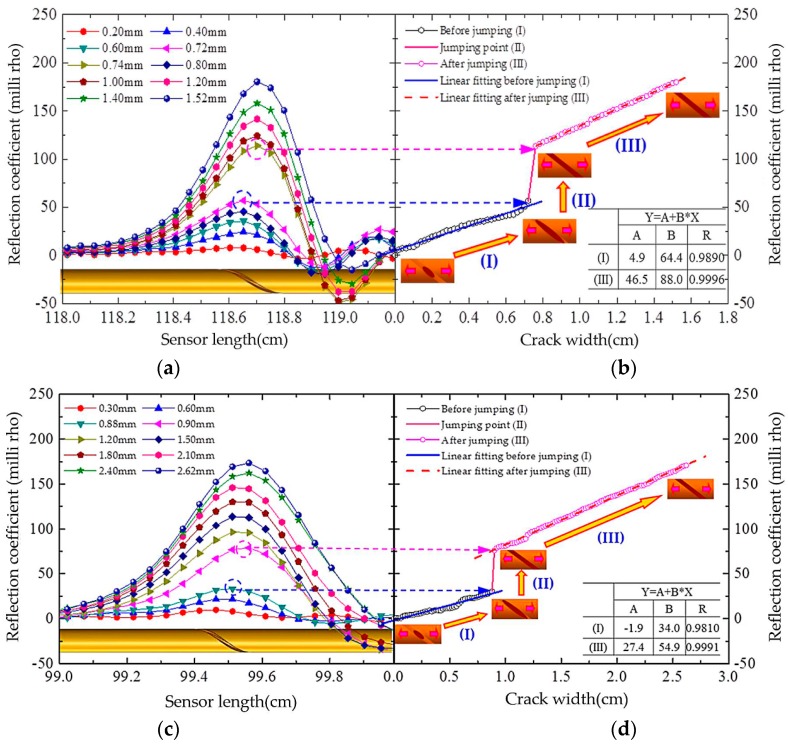
Test results: (**a**) reflection coefficient distribution along Sensor-I; (**b**) reflection coefficient variations with crack width of Sensor-I; (**c**) reflection coefficient distribution along Sensor-II; and (**d**) reflection coefficient variations with crack width of Sensor-II.

Each prototype sensor mounted on the test system was elongated at a step of 0.02 mm, and the response waveforms were captured by the VNA. It was observed during the experiment that the shallow spiral groove was gradually separated from 0 mm up to 1.52 mm of Sensor-I and 2.62 mm of Sensor-II with the increase of the load level. Figure 9a,c presents the resulting waveforms in terms of the reflection coefficient distribution of the two tested sensors. For the convenience of quantitative analysis, their reflection coefficient variations with crack width are also shown in Figure 9b,d. It is apparent that the separating process of the spiral groove goes through three stages under tensile loads. In stage I, the spiral groove starts to separate when an initial strain occurs. Then, the crack develops along the helical direction of spiral groove with gradual increases from 0, up to 0.5 turns. As shown in Figure 9b,d, the peak values of the reflection coefficient increase almost linearly with the applied strain during this process. Stage II presents a sharp jump in the relationship curve between the reflection coefficient and the crack width since the crack of the spiral groove stops developing along its helical direction and experiences a sudden extension along the axial orientation of the cable. Starting from the stage III, the crack width linearly increases with the applied displacement. Consequently, the peak values of the reflection coefficient are linearly related to crack width in this stage. It could be seen that the sensitivity in stage III is higher than that in stage I for both Sensor-I and Sensor-II. The comparison between Sensor-I and Sensor-II also show that Sensor-I is more sensitive to crack than Sensor-II. It means that a higher sensitivity can be obtained if the spiral groove interval is designed to be smaller.

**Figure 10:**
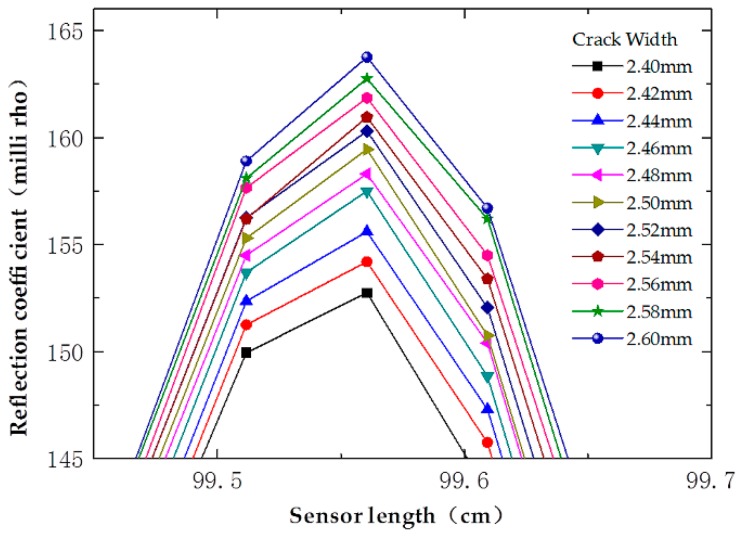
Relationship of the reflection coefficient with crack width (varying from 2.40 mm to 2.60 mm) of Sensor-II.

To verify the resolution of the prototype sensor, response waveforms of Sensor-II are partially enlarged and presented in Figure 10, under different crack widths that vary from 2.40 mm to 2.60 mm, with an increment of 0.02 mm. It can be concluded that the prototype sensor can distinguish the crack width of 0.02 mm.

### 5.2. Multiple Crack Sensing Test

Based on transmission line theory, any separation of the spiral groove on the designed sensor will cause EM wave reflection. The sensing cable, thus, can function as a group of many sensing elements when it suffers from multiple cracks. Therefore, it is important to validate the spatial resolution of the sensor for multiple cracks detecting. Table 3 lists the spatial resolution and parameters, obtained from the test of two kinds of sensors with multi-spiral grooves fabricated.

**Figure 11:**
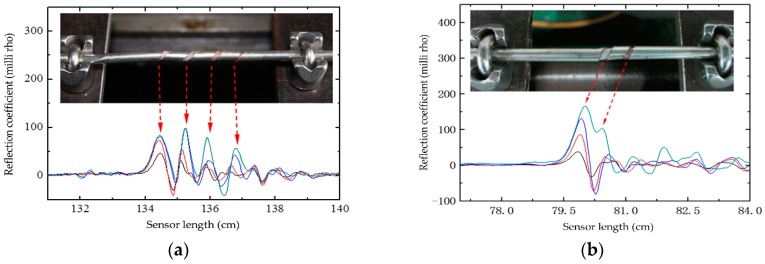
Reflection coefficient distribution along the sensor: (**a**) Sensor-III; and (**b**) Sensor-IV.

In this test, the rise time of the incident impulse signal launched by the VNA is 6 ps, and the bandwidth of the tested sensor is 12 GHz. Sensor-III and Sensor-IV mounted on the test system were elongated at a step of 0.02 mm respectively, and their reflection coefficient distributions along the sensor captured by the VNA are presented in Figure 11. It is seen in Figure 11a that four peaks of the waveform corresponding to the spiral grooves of Sensor-III are clearly distinguished. When the spiral groove interval is 3.0 mm, as in Sensor-IV, its reflection peaks can also be distinguished but not as distinct as Sensor-III. From Figure 11b, the adjacent peaks will overlap if the spiral groove interval is less than 3.0 mm, resulting in a minimum spatial resolution of 3.0 mm. Consequently, the theoretical spatial resolution (2.98 mm) calculated by Equation agrees well with the experiment result.

## 6. Experiment Validation on RC Members

**Figure 12:**
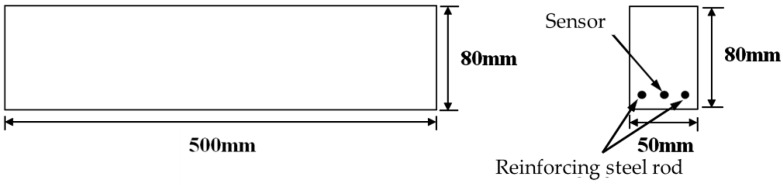
Geometric configuration of the specimen.

To confirm the effectiveness of the prototype sensor in practical applications, a three-point bending test and a four-point bending test were conducted separately on two identical small-scale RC beams with an embedded coaxial prototype crack-detecting sensor. The three-point bending test is expected to explore the single crack detecting ability of the prototype sensor, and the main objective of the four-point bending test is to validate the multiple cracks sensing ability. The concrete beam was 500 mm long with a 150 mm × 250 mm rectangular cross-section. Two steel rods of 8.00 mm diameter were used to reinforce the beam and were placed at a distance of 15 mm from the bottom surface and 60 mm apart symmetrically about the centerline of the cross-section. The sensor made of semi-rigid SFT-50-3 coaxial cable has a diameter of 3.60 mm, then placed on the middle-line of the specimen at the same height of the steel rods. The geometric configuration of the cross-section of the beam is shown in Figure 12.

**Figure 13:**
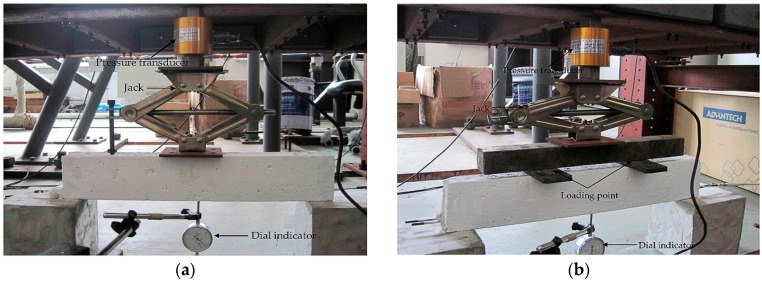
Test setup: (**a**) three-point bending test; and (**b**) four-point bending test.

The concrete beam specimens were cast using the commercial C25 concrete mix. A wooden mold was used for casting the sample. During the casting, the sensing cable was held in place by two spring clamps in which a taut tension was exerted to prevent the cable from sagging. The specimens were cured for 28 days before testing. Then step loading on the RC beam was conducted by a reaction frame and a pressure transducer, as well as a jack. A dial indicator was also instrumented at the mid-span of the beam to measure deflections. A strip of narrow steel plate was added in between the loading wedge and the specimen, to prevent a local cracking of the specimen at the point of the load application in the three-point bending test. Photographs showing the test setups of three-point bending test and four-point bending test are given in Figure 13. A quasi-static monotonic bending load with a step load increment of 0.2 kN was applied up to the failure of the specimen. At every load step, the response ETDR signal waveforms of the embedded sensor were acquired using a VNA, and a crack width gauge measures the crack width.

### 6.1. Results Analysis of a Three-point Bending Test

**Figure 14:**
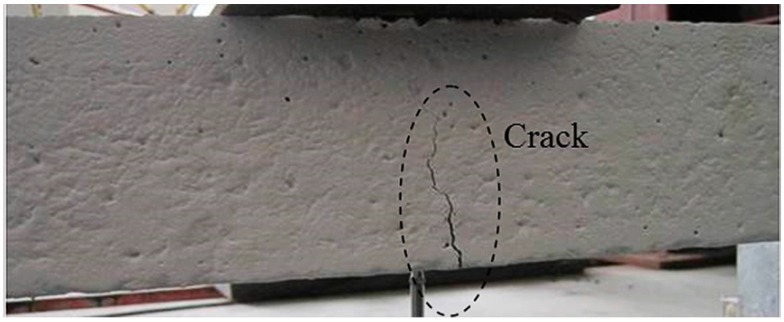
Photograph of the specimen failed with single crack damage.

**Figure 15:**
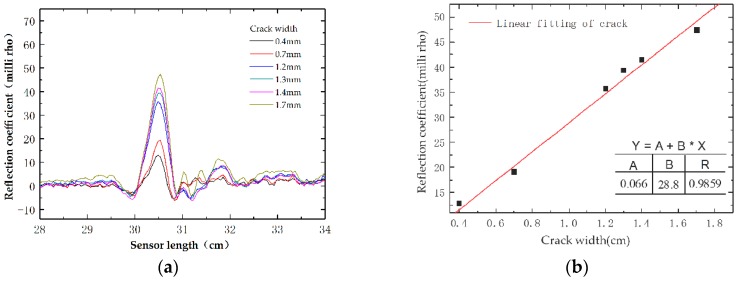
Test results of the three-point bending test: (**a**) experimental reflection waveform; and (**b**) the correlation between the reflection coefficient and crack width.

An initial crack with 0.4 mm width occurs at 3.6 kN of applied load during the test; then the crack grows as the load increases. The maximum crack width recorded at specimen failure is 1.7 mm. A photograph of the failed specimen and the corresponding ETDR signal responses of the embedded sensing cable regarding reflection coefficient are presented in Figure 14 and Figure 15, respectively. The crack damage induced on the specimen near the mid-span of the beam (Figure 14), is clearly indicated by a spike in the response waveform shown in Figure 15a. Note that the reflection coefficient outside the mid-span region is all within 3~10 milli-rho, which is due to small elongation in the non-cracking area. Figure 15b indicates that the peak values of the reflection coefficient increase almost linearly with respect to the crack width. The test results verify that the prototype sensor can detect the location and relative magnitude of the crack damage of a structure after calibration.

### 6.2. Results Analysis of a Four-Point Bending Test

**Figure 16:**
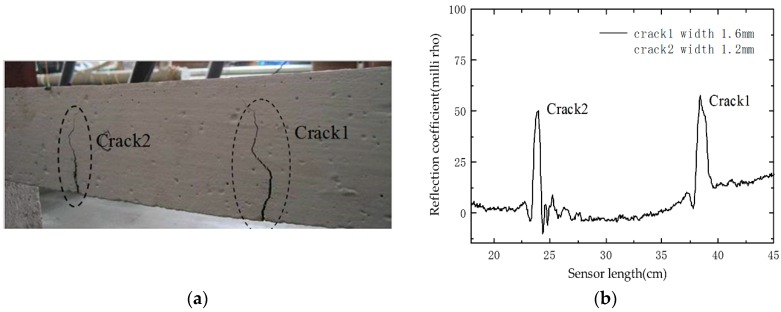
Test results: (**a**) crack patterns of the tested beam; and (**b**) the corresponding reflection waveform.

**Figure 17:**
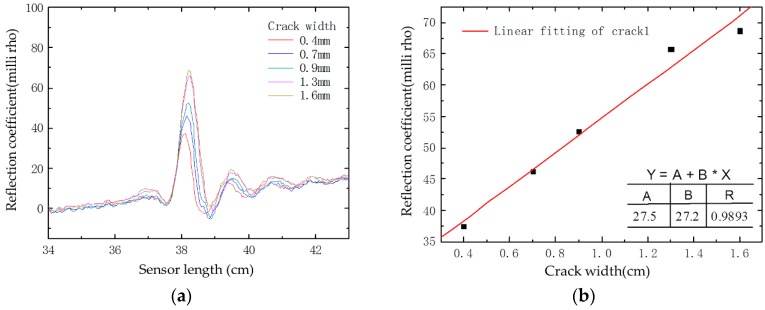
Test results of a four-point bending test: (**a**) experimental reflection waveform; and (**b**) correlation between the reflection coefficient and the crack width.

Under progressive loading, two dominant cracks appeared at two loading points in the four-point bending test as presented in Figure 16a. Two spikes corresponding to the cracks’ locations capture and clearly identify the cracks from the reflection waveform as shown in Figure 16b. Therefore, the embedded sensor is capable of detecting multiple cracks. Figure 17 indicates the waveform of crack1 as an example. The crack width grows gradually from 0.4 mm to 1.6 mm with the increase of applied loading. From Figure 17a, we can observe that reflection coefficient changes generally as the crack develops. The peak values of the reflection coefficient induced by crack1 are plotted in Figure 17b, acting as a function of crack width. It shows that the peak values increase linearly as the crack width enlarges. These results imply that the prototype sensor has the ability of multiple crack monitoring.

## 7. Conclusions

This paper reports a distributed crack sensor using a coaxial cable based on the ETDR sensing principle. The prototype sensor designed with a shallow helical groove on the outer surface of the coaxial cable is successfully fabricated by a CNC milling machine. The sensor is expected to have lower signal attenuation than the state-of-the-art sensors, but a comparison test needs to be implemented in future work. The numerical simulations explored the correlation between the sensor performances and their major design parameters. It concludes that a higher peak value of the reflection coefficient corresponds to a smaller interval of the spiral groove, and a higher spatial resolution might be obtained by reducing the rise time of the incident impulse signal. The sensor performance was investigated through calibration tests. In the test, the reflection waveform measured with a cable sensor correlates well with the location and width of a crack. Results demonstrate that the prototype sensor is capable of detecting cracks of 0.02 mm and sensing multiple cracks along the cable with a minimum spatial resolution is 3.0 mm. Finally, a feasibility study was also conducted by using the prototype sensor to detect the presence of load-induced crack damages and identify their locations in a RC member. It confirms that the prototype sensor can successfully be used to monitor the initiation and development of cracks on a structure up to its failure.

## Figures and Tables

**Table 1:** Parameters of SFT-50-3 coaxial cable.

Diameter of Inner Conductor	Diameter of Outer Conductor	Thickness of Outer Conductor	Characteristic Impedance	Dielectric Constant
0.92 mm	3.60 mm	0.30 mm	50 Ω	2.10

**Table 2:** Prototype sensors for a sensitivity test.

Sensor	Length of Sensor	Turns of Spiral Separation	Initial Position of the Spiral Groove	Spiral Groove Interval
Sensor-I	150 cm	1.0	118.5 cm	3.0 mm
Sensor-II	150 cm	1.0	99.5 cm	5.0 mm

**Table 3:** Types of prototype sensors for the spatial resolution test.

Sensor	Length of Sensor	Turns of Spiral Separation	Initial Position of the Spiral Groove	Spiral Groove Interval
Sensor-III	150 cm	4.0	134.0 cm	5.0 mm
Sensor-IV	150 cm	2.0	79.5 cm	3.0 mm
